# Supplementary material for: Robust use of phenotypic heterogeneity at drug target genes for mechanistic insights: application of cis-multivariable Mendelian randomization to GLP1R gene region
Source: Genet Epidemiol. Author manuscript; Available in PMC 2024 Jul 10. (PMC7616158; doi:10.1002/gepi.22551)
Supplement: Supplementary material [file EMS196988-supplement-Supplementary_material.pdf]

Robust use of phenotypic heterogeneity at drug target genes for mechanistic insights: application of *cis*-multivariable Mendelian randomization to *GLP1R* gene region

Supplementary Material

The robust PC-GMM method

Linear model with dimension-reduced instruments

Let $X = (X_1, \ldots, X_K)'$ denote a K-vector of risk factors, Y an outcome, and $Z = (Z_1, \ldots, Z_M)'$ an M-vector of genetic variants. Our focus is on a *cis*-gene analysis where genetic variants from a single region are in highly structured correlation. Let Λ denote the $M \times L$ matrix where its columns are the first L principal components ($K \leq L < M$) of a weighted sample correlation matrix of genetic variants.[1] We consider the following linear IV model with homoscedastic errors

$$Y = \omega + \theta_0' X + \alpha'(\Lambda' Z) + U \quad (1)$$

$$X = \psi + \gamma'(\Lambda' Z) + V \quad (2)$$

where $E[U|\Lambda' Z] = 0$, $E[V|\Lambda' Z] = 0$, $E[U^2|\Lambda' Z] = \sigma_U^2$, $E[VV'|\Lambda' Z] = \Sigma_V$, and $(\omega, \psi, \gamma, \theta_0)$ are unknown parameters. We assume α is a mean-zero random effect that is uncorrelated with all other variables. We are interested in estimation and inference on the K-vector of risk factor effects on the outcome θ_0, using only two-sample summary data that is often made publicly available.

For each variant m and risk factor k, we have access to estimates $\widehat{\beta}_{X_{km}}$ and standard errors $\sigma_{X_{km}}$ from univariable X_k on Z_m linear regressions from an n_X-sized sample, and from a non-overlapping n_Y-sized sample, we observe measured associations $(\widehat{\beta}_{Y_m}, \sigma_{Y_m})$ from univariable Y on Z_m linear regressions. Both random samples are drawn from the joint distribution of (Y, X, Z). We assume knowledge of an $M \times M$ genetic correlation (or linkage disequilibrium) matrix ρ, where its (m_1, m_2)-th element $\rho_{m_1 m_2}$ denotes the correlation between the m_1-th and m_2-th genetic variant. Finally, we also assume knowledge of a $K \times K$ risk factor correlation matrix τ, where its (k_1, k_2)-th element $\tau_{k_1 k_2}$ denotes the correlation between X_{k_1} and X_{k_2}.

Substituting (2) into (1), we have $Y = (\omega + \theta_0'\psi) + (\gamma\theta_0 + \alpha)'(\Lambda' Z) + (U + \theta_0' V)$. Thus, $Cov(\Lambda' Z, Y) = Var(\Lambda' Z)(\gamma\theta_0 + \alpha)$, which leads to a model

$$\Gamma = \gamma\theta_0 + \alpha, \text{ where } \alpha \sim N(0_{L\times 1}, I_L \kappa^2 n_Y^{-1}) \quad (3)$$

where Γ is the L-vector of coefficients from a population multivariable regression of Y on $\Lambda' Z$, and γ is the $L \times K$ matrix such that its k-th column γ_k is the L-vector of coefficients from a population multivariable regression of X_k on $\Lambda' Z$. The random effects α are mutually uncorrelated, and assumed to be normally distributed scaled up to an unknown overdispersion variance parameter κ^2.

Proposition 1 (Two-sample summary data associations). *Using the two-sample univariable summary data described above, we can construct multivariable estimates* $(\widehat{\Gamma}, \widehat{\Sigma}_\Gamma)$ *and* $(vec(\widehat{\gamma}), \widehat{\Sigma}_\gamma)$*, such that*

$$\begin{bmatrix} \sqrt{n_Y}(\widehat{\Gamma} - \gamma\theta_0) \\ \sqrt{n_X}(vec(\widehat{\gamma}) - vec(\gamma)) \end{bmatrix} \xrightarrow{D} N\left(\begin{bmatrix} 0_{L\times 1} \\ 0_{LK\times 1} \end{bmatrix}, \begin{bmatrix} \Sigma_\Gamma + I_L\kappa^2 & 0_{L\times LK} \\ 0_{LK\times L} & \Sigma_\gamma \end{bmatrix} \right),$$

$\widehat{\Sigma}_\Gamma - \Sigma_\Gamma \xrightarrow{P} 0$, *and* $\widehat{\Sigma}_\gamma - \Sigma_\gamma \xrightarrow{P} 0$, *as* $(n_X, n_Y) \to \infty$.

Conditional F-statistics

We require some additional notation to construct conditional F-statistics in our setting of dimension-reduced genetic associations. Let $\widehat{\Sigma}_{\gamma,k}$ denote the matrix $\widehat{\Sigma}_\gamma$ that is rearranged such that the k-th column is moved left to be the first column, and the k-th row is moved up to be the first row. Let $\widehat{\gamma}_{-k}$ denote the $L \times (K-1)$ matrix equal to $\widehat{\gamma}$ without the k-th column, let γ_{-k} denote the $L \times (K-1)$ matrix equal to γ without the k-th column, and let $h(\delta) = [I_L \quad -I_L \otimes \delta']$ denote the $L \times LK$ matrix where δ is a $(K-1)$-vector of unknown parameters, and where $\otimes$ denotes the Kronecker product.

Then, for any risk factor k, we consider conditional F-statistics[2] given by

$$F_{k|-k} = \frac{n_X}{L-K+1} \min_\delta \{ (\widehat{\gamma}_k - \widehat{\gamma}_{-k}\delta)' [h(\delta)\widehat{\Sigma}_{\gamma,k} h(\delta)']^{-1} (\widehat{\gamma}_k - \widehat{\gamma}_{-k}\delta) \}.$$

Proposition 2 (Conditional F-statistics). *For any risk factor k, under the null hypothesis H_{0k} : $\gamma_k - \gamma_{-k}\delta = 0$ uniquely at some $\delta = \delta_0$, $F_{k|-k}(L-K+1) \xrightarrow{D} \chi^2_{L-K+1}$ as $n_X \to \infty$.*

Under the null hypothesis $H_{0k} : \gamma_k - \gamma_{-k}\delta = 0$ uniquely at some $\delta = \delta_0$, Proposition 2 shows that the statistic $F_{k|-k}(L-K+1)$ should behave like a χ^2_{L-K+1} random variable in large samples. This allows us to perform a test of no phenotypic heterogeneity for any risk factor k, with a rejection of the null hypothesis H_{0k} suggesting evidence for phenotypic heterogeneity.

Robust PC-GMM: 2-step estimation of θ_0 and κ^2

By Proposition 1, we have $E[\widehat{\Gamma} - \widehat{\gamma}\theta_0] = 0_{L\times 1}$, so that there are L moment equations which describe θ_0. To identify θ_0, we require the usual rank condition that the column rank of γ is at least K.

Let $\widehat{g}(\theta) = \widehat{\Gamma} - \widehat{\gamma}\theta$ denote an L-vector of estimating functions. Then, using Proposition 1, an estimator of the variance of $\widehat{g}(\theta_0)$ is given by $\widehat{\Omega}(\theta_0, \kappa^2) = n_Y^{-1}(\widehat{\Sigma}_\Gamma + I_L\kappa^2) + n_X^{-1}\varphi(\theta_0)\widehat{\Sigma}_\gamma\varphi(\theta_0)'$, where $\varphi(\theta) = \theta' \otimes I_L$. Thus, under knowledge of κ^2, we consider $\widehat{Q}(\theta, \kappa) = \widehat{g}(\theta)'\widehat{\Omega}(\theta, \kappa^2)^{-1}\widehat{g}(\theta)$ as the oracle continuously-updating GMM[3,4] criterion function based on the optimal weighting matrix.

Proposition 3 (Oracle GMM estimation)**.** *Under regularity conditions, the oracle GMM estimator* $\bar{\theta} = \arg\min_\theta \widehat{Q}(\theta, \kappa^2)$ *is consistent for* θ_0*, and is asymptotically distributed* $\sqrt{n_Y}(\bar{\theta} - \theta_0) \xrightarrow{D} N(0_{K\times 1}, \Sigma_\theta)$ *where* $\Sigma_\theta = (\gamma'\Omega^{-1}\gamma)^{-1}$ *where* $\Omega = \Sigma_\Gamma + I_L\kappa^2 + c\varphi(\theta_0)\Sigma_\gamma\varphi(\theta_0)'$ *and* $n_X^{-1}n_Y \to c$*, as* $(n_X, n_Y) \to \infty$*.*

Proposition 3 implies that the standard errors of $\bar{\theta}$ need to account for the extra uncertainty due to the random direct effects, which is represented by the overdispersion variance parameter κ^2. However, consistent estimation of θ_0 is still possible by ignoring the extra uncertainty. In particular, $\widehat{\theta}_1 = \arg\min_\theta \widehat{Q}(\theta, 0)$ is a consistent GMM estimator of θ. We can then use this preliminary estimator $\widehat{\theta}_1$ to pin down κ^2.

Noting that $\widehat{Q}(\widehat{\theta}_1, \kappa^2) \xrightarrow{D} \chi^2_{L-K}$, an estimator $\widehat{\kappa}^2$ of κ^2 solves the estimating equation $\widehat{Q}(\widehat{\theta}_1, \widehat{\kappa}^2) - (L-K) = 0$. Then, the 2-step estimator of θ_0 is given by $\widehat{\theta} = \arg\min_\theta \widehat{Q}(\theta, \widehat{\kappa}^2)$. Finally, it is straightforward to estimate the standard errors of $\hat{\theta}$ based on Proposition 3. For the k-th risk factor, the estimated standard errors are given by the square root of the (k, k)-th element of the matrix $(\widehat{\gamma}'\widehat{\Omega}(\widehat{\theta}, \widehat{\kappa}^2)^{-1}\widehat{\gamma})^{-1}$.

Finally, for the unrobust version of the PC-GMM method, a test of overidentifying restrictions[5] (henceforth, heterogeneity test) is a useful way to assess the coherency of evidence over all instruments. Assuming that θ_0 is identified (so that there are at least K distinct valid instruments in the vector $\Lambda'Z$), if one of the instruments is invalid, then $\widehat{g}(\theta_0)$ no longer has mean zero, and therefore we would expect the GMM criterion $\widehat{Q}(\theta_0, 0)$ to deviate further away from 0. This is the intuition behind the heterogeneity test.

More formally, by very similar arguments used in Proof of Proposition 2, it can be shown under no overdispersion heterogeneity ($\kappa^2 = 0$) that $\min_\theta \widehat{Q}(\theta, 0) \xrightarrow{D} \chi^2_{L-K}$. Therefore, we can compute a heterogeneity test by comparing the statistic $\widehat{Q}(\widehat{\theta}, 0)$ against a relevant critical value from the χ^2_{L-K} distribution. Let c_α denote the $(1-\alpha)$-th quantile of the χ^2_{L-K} distribution. If $\widehat{Q}(\widehat{\theta}, 0) > c_\alpha$, then we reject the null hypothesis of instrument coherency for an α-level test.

Further empirical results

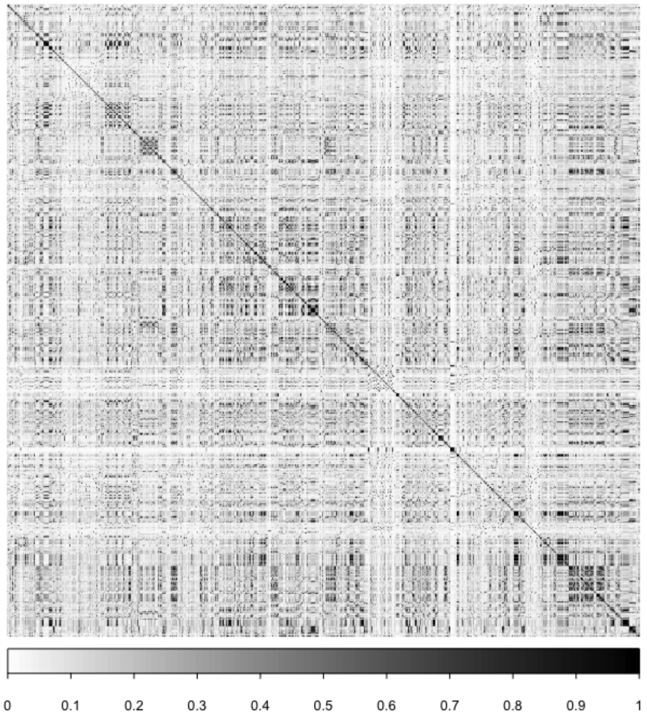

Supplementary Figure S1. The absolute values of the correlation matrix of 851 genetic variants in *GLP1R*.

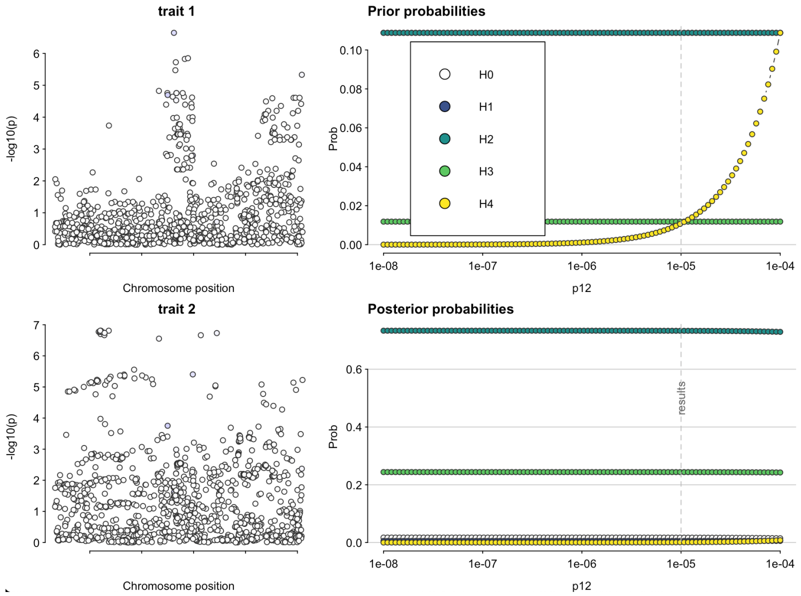

Supplementary Figure S2. Sensitivity of coloc results to the choice of prior p_{12} on the shared causal variant hypothesis.

PC-GMM method	risk factor	estimate	95% CI	p-value	# PCs	het. test	cond. F-stat.
robust (99.9%)	BMI	0.769	0.126, 1.412	0.019	54	-	2.304
	T2D	0.104	-0.068, 0.276	0.236			2.796
unrobust (99.9%)	BMI	0.819	0.324, 1.313	0.001		<0.001	-
	T2D	0.026	-0.102, 0.153	0.694			-
robust (99%)	BMI	1.146	0.292, 2.000	0.009	25	-	3.516
	T2D	-0.012	-0.241, 0.217	0.917			4.475
unrobust (99%)	BMI	1.221	0.609, 1.833	<0.001		<0.001	-
	T2D	-0.105	-0.260, 0.050	0.185			-
robust (95%)	BMI	2.427	-0.053, 4.907	0.055	12	-	3.224
	T2D	-0.176	-0.613, 0.261	0.431			3.228
unrobust (95%)	BMI	2.519	1.293, 3.745	<0.001		0.008	-
	T2D	-0.394	-0.657, -0.130	0.003			-

Supplementary Table S1. PC-GMM results: Genetically-predicted multivariable BMI and T2D effects on CAD risk. The percentages next to the PC-GMM method indicate the percentage of weighted genetic variation explained in *GLP1R* by the number of principal components used.

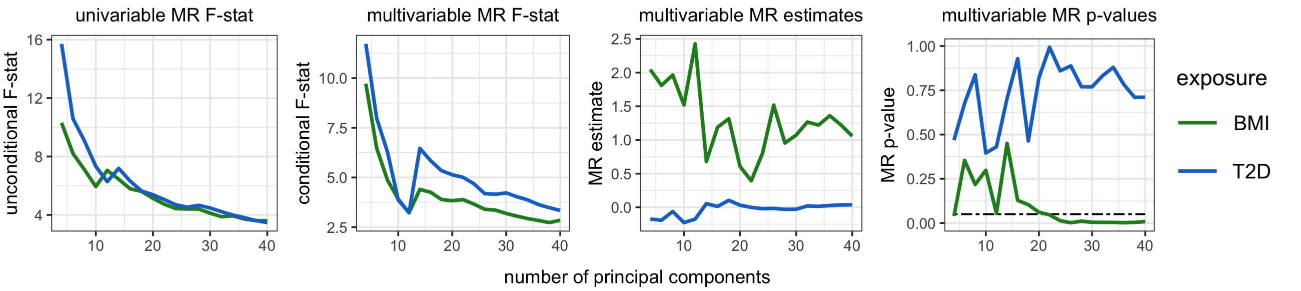

Supplementary Figure S3. Robust PCA-GMM estimates of genetically-predicted effects of BMI and T2D liability on CAD risk when using different numbers of principal components.

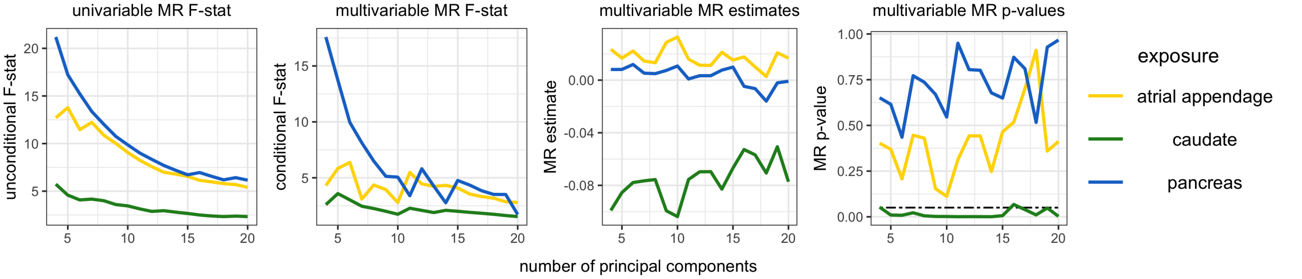

Supplementary Figure S4. Robust PCA-GMM estimates of genetically-predicted effects of GLP1R expression in different tissues on CAD risk when using different numbers of principal components.

Further simulation results

Sensitivity to mis-specifying trait correlations

We consider the impact of mis-specifying the true trait correlations ρ in the same simulation design discussed in the main text, with $\xi = 1$ (phenotypic heterogeneity; all 3 risk factors have 5 distinct causal variants) and $\kappa^2 = 0.5$ (moderate overdispersion heterogeneity). Supplementary Figure S3 shows the impact on type I error (relating to $\theta_2 = 0$) and power (relating to $\theta_1 = -1/3$ and $\theta_3 = 1/3$) of specifying trait correlations $\widehat{\rho}$ instead of ρ.

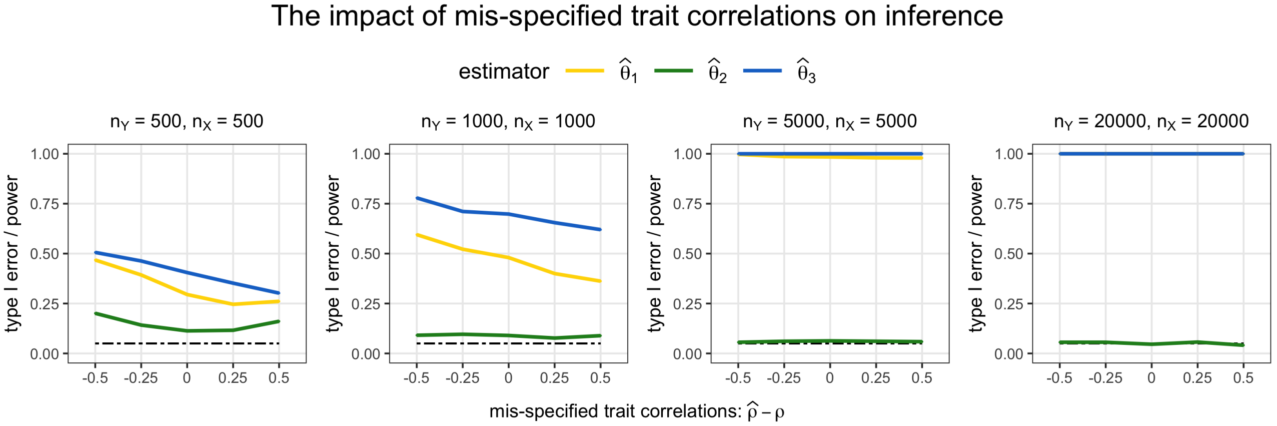

Supplementary Figure S5. Type I error/power varying with mis-specification of trait correlations. The true causal effect is $(\theta_1, \theta_2, \theta_3)' = (-1/3, 0, 1/3)'$.

From Supplementary Figure S3, we find that the results were not sensitive to mis-specification of trait correlations for large enough sample sizes. For small samples ($n_X = n_Y = 500$), mis-specification of trait correlations does appear to harm an already inflated type I error rate. Interestingly, the specified trait correlations also appear to impact the power properties: under-estimating trait correlations ($\widehat{\rho} < \rho$) appeared to be less harmful in terms of power compared with over-estimating the correlations. Although, more generally, the impact on power may depend on several other model parameters, such as the direction of the true trait correlations ρ and the direction of the causal effect $\theta = (\theta_1, \theta_2, \theta_3)'$.

Performance under weak instruments

To investigate the performance of the robust PC-GMM method under weak instruments, under the same simulation design discussed in the main text, we set the sample size at $n = 1000$, and we

changed the causal variant effects on the risk factor:

$$\begin{aligned} X_1 &= \mu \sum_{m=1}^{5} Z_m + V_1 \\ X_2 &= \mu\xi \sum_{m=6}^{10} Z_m + \mu(1-\xi) \sum_{m=11}^{15} Z_m + V_2 \\ X_3 &= \mu \sum_{m=11}^{15} Z_m + V_3, \end{aligned}$$

where the errors (V_1, V_2, V_3) were jointly normally distributed with mean zero such that $var(V_k) = 1$ and $cov(V_k, V_l) = 0.3$ for $k, l \neq k \in \{1, 2, 3\}$. Here, μ represents the unconditional instrument strength, and ξ represents phenotypic heterogeneity for risk factors 2 and 3. When $\xi = 0$, the variants effects on risk factors 2 and 3 are collinear, and when $\xi = 1$, all three risk factors have distinct causal variants. As in the main text, we chose the number of principal components to explain 99.9% of variation in a weighted genetic correlation matrix.

First, for the case of no overdispersion heterogeneity, we reproduce plots analogous to Figure 3 of the main text, but now instead of varying the sample size, we vary the parameter μ. Supplementary Figure 6 presents estimation and inference results for this case.

Supplementary Figure 6 highlights that the source of weak instrument bias has an important impact on estimation and inference. Biased estimation can be expected if either μ is close to 0 (unconditional instrument strength is low) or if ξ is close to 0 (i.e. conditional instrument strength is close to 0). In the case of this simulation exercise, there is a bias toward the null of no effect for each non-zero risk factor effect (θ_1 and θ_3), and an upward bias for the null effect θ_2. This upward bias for the null effect disppears when there is large enough phenotypic heteogeneity (ξ is close to 1) even for low values of unconditional instrument strength μ.

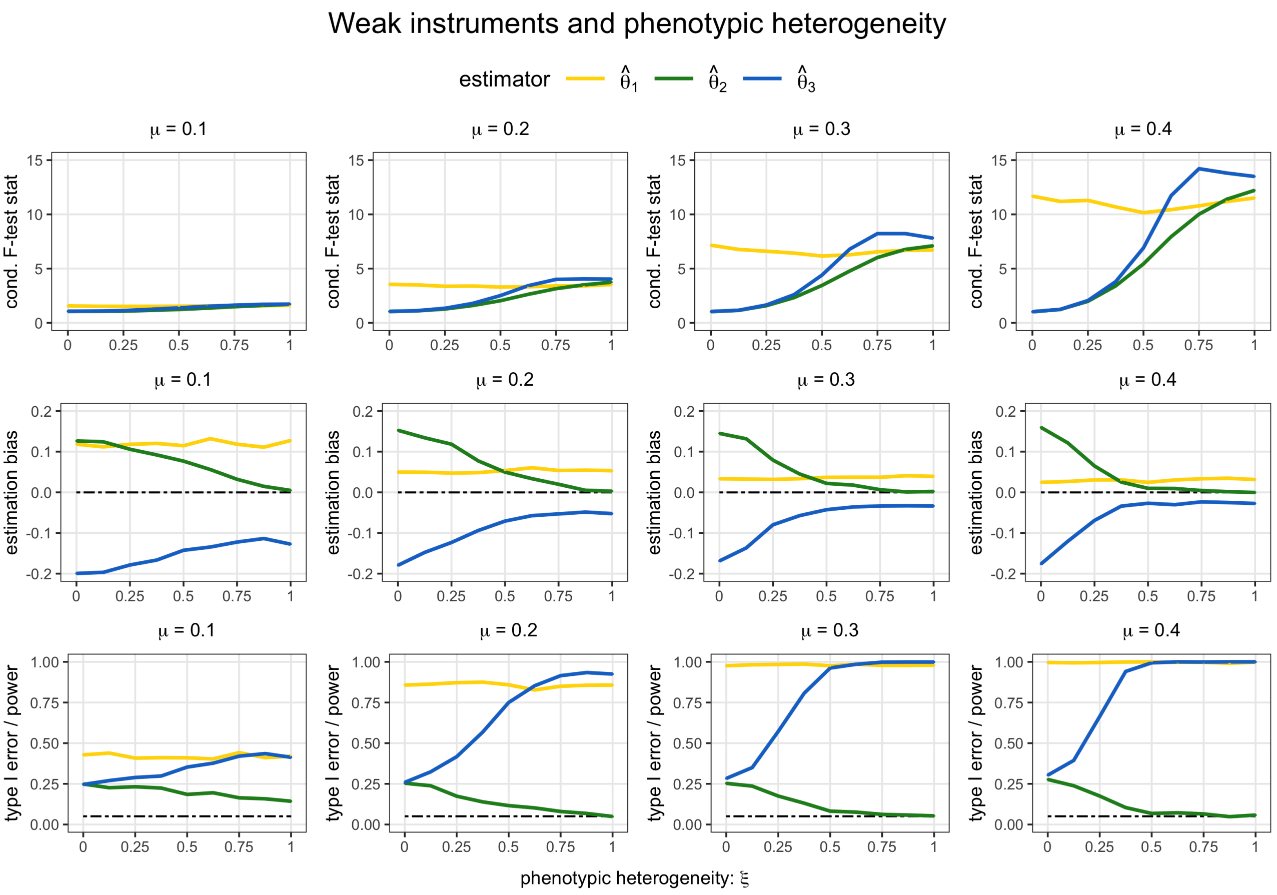

Supplementary Figure S6. Estimation bias and inference using robust PC-GMM under varying phenotypic heterogeneity ξ, varying unconditional instrument strength μ, and no overdispersion heterogeneity ($\kappa^2 = 0$). The true parameter values were $\theta_1 = -1/3$, $\theta_2 = 0$, and $\theta_3 = 1/3$. The nominal size of all tests was 0.05.

For inference, a lack of conditional instrument strength appears to harm size performance more than a lack of unconditional instrument strength. For example, for the case of $\mu = 0.2$ and $\xi = 1$, conditional F-statistics are just over 4, but the type I error rate (for risk factor 2) is exactly equal to the nominal size 0.05. Overall, our simulation results would suggest that we could be less concerned about the impact of weak instrument bias on inference if the conditional F-statistics are not too low and close to unconditional F-statistics (suggesting a scenario where ξ is close to 1).

Supplementary Figure 7 presents results for the case where $\xi = 1$ (all risk factors have distinct causal variants), and under overdispersion heterogeneity in genetic variant–outcome associations (higher values of κ^2 indicate greater overdispersion heterogeneity). The results further illustrate that the type I error rate may be close to the nominal level if there is phenotypic heterogeneity and as long as the unconditional instrument strength is not too low ($\mu \geq 0.2$).

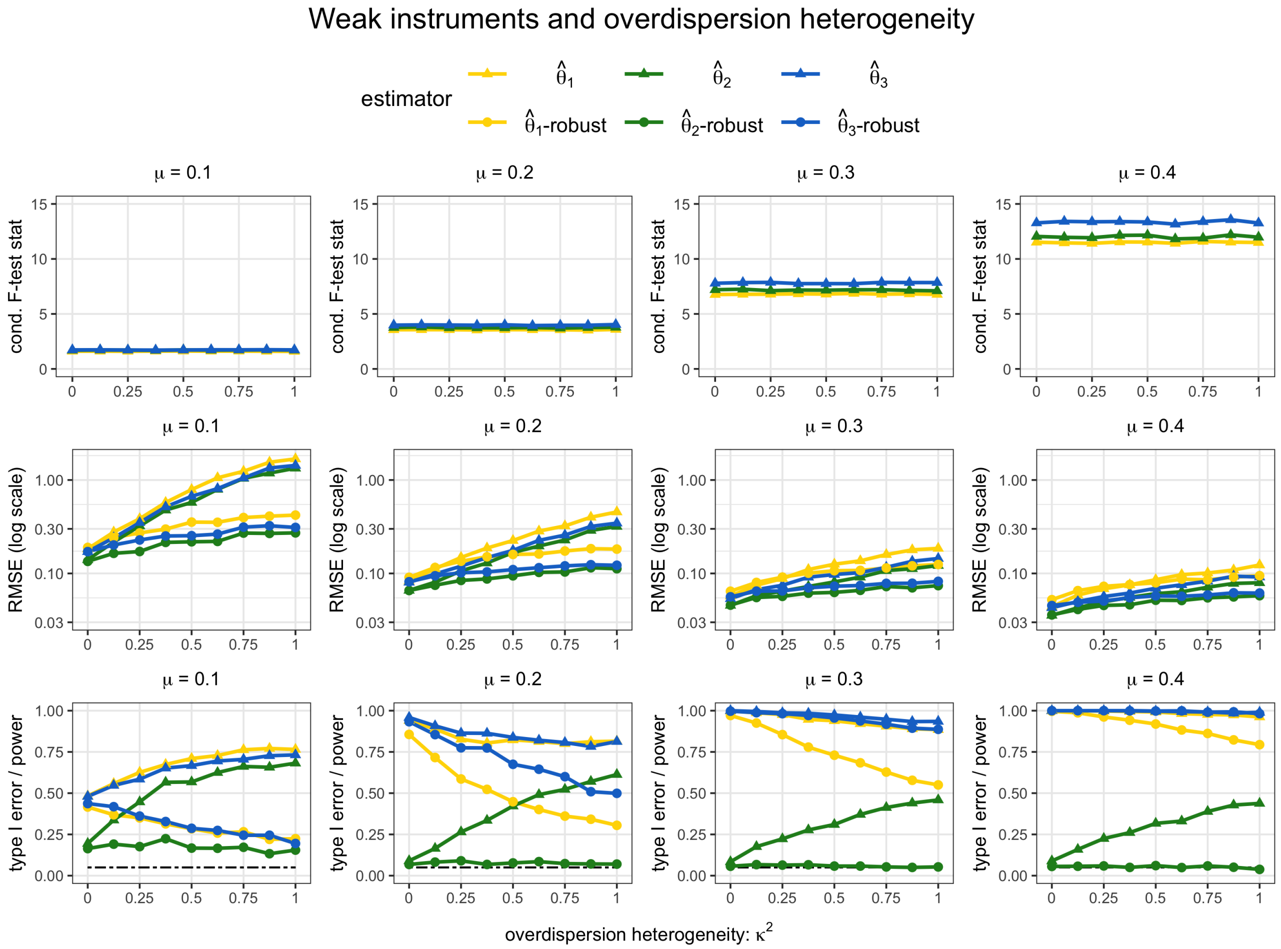

Supplementary Figure S7. RMSE and type I error/power under phenotypic heterogeneity ($\xi = 1$), varying overdispersion heterogeneity ($0 \leq \kappa^2 \leq 1$), and varying unconditional instrument strength μ. The nominal size of all tests was 0.05. The true parameter values were $\theta_1 = -1/3$, $\theta_2 = 0$, $\theta_3 = 1/3$. For each risk factor k, $\hat{\theta}_k$-robust indicates the results using robust PC-GMM, and $\hat{\theta}_k$ indicates the results using unrobust PC-GMM (which assumes $\kappa^2 = 0$).

Varying the number of principal components

Supplementary Figure 8 presents simulation results under the same design discussed in the main text, and where we vary the number of principal components used to instrument the risk factors. The sample size set at $n = 1000$, and the overdispersion heterogeneity parameter was set at $\kappa^2 = 0.75$. Here, we see that the optimal number of principal components to maximise conditional F-statistics is not necessarily the best for inference. For example, when $\xi = 1$ the conditional F-statistics for all risk factors are higher when using 20 principal components compared with using 30, but while the type I error rate is the same for risk factor 2 the power to detect the non-zero effects for risk factors 2 and 3 is higher when using 30 principal components.

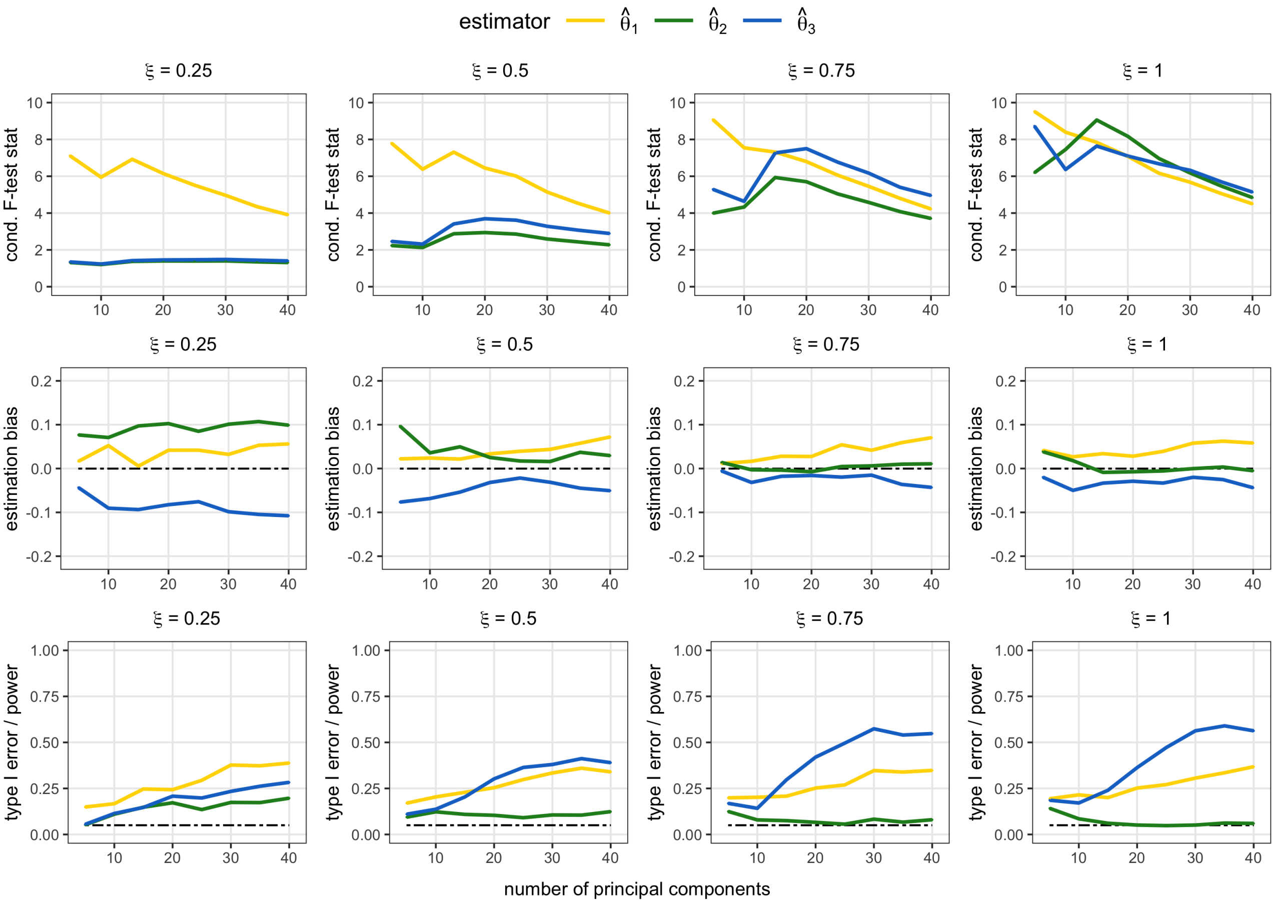

Supplementary Figure S8. Estimation bias and inference using robust PC-GMM under varying phenotypic heterogeneity ξ and the number of principal components used as instruments. The overdispersion heterogeneity parameter was set at $\kappa^2 = 0.75$, and the sample size was set at $n = 1000$. The true parameter values were $\theta_1 = -1/3$, $\theta_2 = 0$, and $\theta_3 = 1/3$. The nominal size of all tests was 0.05.

Appendix

Proof of Proposition 1

Asymptotic distributions of $\widehat{\Gamma}$ and $\widehat{\gamma}$

From Equations (1)–(3), we have the reduced form outcome model $Y = \eta + (\gamma\theta_0 + \alpha)'(\Lambda' Z) + \varepsilon$, where $\eta = \omega + \theta_0'\psi$, and $\varepsilon = U + \theta_0' V$, so that $E[\varepsilon|\Lambda' Z] = 0$ and $var(\varepsilon|\Lambda' Z) = \sigma_\varepsilon^2$.

Let $\widehat{\Gamma} = \widehat{var}(\Lambda' Z)^{-1}\widehat{cov}(\Lambda' Z, Y)$ be the estimated coefficient of a multivariable linear regression of Y on $\Lambda' Z$ with a constant term included. Note that $E[\widehat{\Gamma}] = \gamma\theta_0$, $\sqrt{n_Y}\big(\widehat{cov}(\Lambda' Z, Y) - cov(\Lambda' Z, Y)\big) \xrightarrow{D} N(0_{L\times 1}, var(\Lambda' Z)\sigma_\varepsilon^2 + var(\Lambda' Z) I_L \kappa^2 var(\Lambda' Z)')$ by a central limit theorem (CLT), and $\widehat{var}(\Lambda' Z) \xrightarrow{P} var(\Lambda' Z)$ by the weak law of large numbers (WLLN).

Hence, by Cramer's theorem, $\sqrt{n_Y}(\widehat{\Gamma}-\gamma\theta_0) \xrightarrow{D} N(0_{L\times 1}, I_L\kappa^2+\Sigma_\Gamma)$ where $\Sigma_\Gamma = var(\Lambda'Z)^{-1}\sigma_\varepsilon^2$, and $\sigma_\varepsilon^2 = var(Y) - cov(\Lambda'Z,Y)'var(\Lambda'Z)^{-1}cov(\Lambda'Z,Y)$.

For each risk factor $k = 1,\ldots,K$, let $\widehat{\gamma}_k = \widehat{var}(\Lambda'Z)^{-1}\widehat{cov}(\Lambda'Z,X_k)$ be the estimated coefficients of a multivariable regression of X_k on $\Lambda'Z$ with a constant term included. By similar arguments to the above, we have $\sqrt{n_X}(\widehat{\gamma}_k-\gamma_k) = var(\Lambda'Z)^{-1}\sqrt{n_X}\widehat{cov}(\Lambda'Z,V_k)+o_P(1)$. Therefore, by a CLT and the Cramer-Wold device, $\sqrt{n_X}(vec(\widehat{\gamma})-vec(\gamma)) \xrightarrow{D} N\big(0_{LK\times 1},\Sigma_\gamma\big)$, where Σ_γ is a $LK\times LK$ variance–covariance matrix.

Under homoscedastic errors, Σ_γ has the following block structure

$$\Sigma_\gamma = \begin{bmatrix} \underset{(L\times L)}{\Sigma_{\gamma,11}} & \underset{(L\times L)}{\Sigma_{\gamma,12}} & . & . & \underset{(L\times L)}{\Sigma_{\gamma,1K}} \\ \underset{(L\times L)}{\Sigma_{\gamma,21}} & \underset{(L\times L)}{\Sigma_{\gamma,22}} & & & . \\ . & & . & & . \\ . & & & . & . \\ \underset{(L\times L)}{\Sigma_{\gamma,K1}} & . & . & . & \underset{(L\times L)}{\Sigma_{\gamma,KK}} \end{bmatrix},$$

where $\Sigma_{\gamma,k_1k_2} = \big(var(\Lambda'Z)^{-1}\big(cov(X_k,X_m) - cov(\Lambda'Z,X_k)'var(\Lambda'Z)^{-1}cov(\Lambda'Z,X_m)\big)\big)$ for any risk factors k_1 and k_2.

Constructing $(\widehat{\Gamma},\widehat{\Sigma}_\Gamma)$ and $(\widehat{\gamma},\widehat{\Sigma}_\gamma)$ from two-sample summary data

We follow the strategy of Wang and Kang (2022).[6] First, we need to calculate principal components of $\widehat{cov}(Z)$. For each variant m, we have $(n_Y\sigma^2_{Y_m}+\widehat{\beta}^2_{Y_m})^{-1} = \widehat{var}(Y)^{-1}\widehat{var}(Z_m)$. Let A_Y be the $M\times M$ matrix with its (m_1,m_2)-th element given by $\rho_{m_1m_2}(n_Y\sigma^2_{Y_{m_1}}+\widehat{\beta}^2_{Y_{m_1}})^{-\frac{1}{2}}(n_Y\sigma^2_{Y_{m_2}}+\widehat{\beta}^2_{Y_{m_2}})^{-\frac{1}{2}}$, so that $A_Y = \widehat{var}(Y)^{-1}\widehat{var}(Z)$. Let Λ be the $M\times L$ matrix with its columns given by first L principal components of A_Y.

Let $b_{Y_m} = (n_Y\sigma^2_{Y_m}+\widehat{\beta}^2_{Y_m})^{-1}\widehat{\beta}_{Y_m} = \widehat{var}(Y)^{-1}\widehat{cov}(Z_m,Y)$, so that $b_Y = (b_{Y_1},\ldots,b_{Y_p})' = \widehat{cov}(Z,Y)\widehat{var}(Y)^{-1}$. Then, $\widehat{\Sigma}_\Gamma = \big(\Lambda'A_Y\Lambda\big)^{-1}\big[1-\big(\Lambda'b_Y\big)'\big(\Lambda'A_Y\Lambda\big)^{-1}\big(\Lambda'b_Y\big)\big] \xrightarrow{P} \Sigma_\Gamma$ by WLLN. Also, note that $\widehat{\Gamma} = \big(\Lambda'A_Y\Lambda\big)^{-1}\Lambda'b_Y$.

We can use a similar strategy for the risk factor model. For each variant m and risk factor k, note that $(n_X\sigma^2_{X_{km}}+\widehat{\beta}^2_{X_{km}})^{-1} = \widehat{var}(X_k)^{-1}\widehat{var}(Z_m)$. For each risk factor k, let A_{X_k} be the $M\times M$ matrix with its (m_1,m_2)-th element given by $\rho_{m_1m_2}(n_X\sigma^2_{X_{km_1}}+\widehat{\beta}^2_{X_{km_1}})^{-\frac{1}{2}}(n_X\sigma^2_{X_{km_2}}+\widehat{\beta}^2_{X_{km_2}})^{-\frac{1}{2}}$, so that $A_{X_k} = \widehat{var}(X_k)^{-1}\widehat{var}(Z)$.

Let $b_{X_{km}} = (n_X\sigma^2_{X_{km}}+\widehat{\beta}^2_{X_{km}})^{-1}\widehat{\beta}_{X_{km}} = \widehat{var}(X_k)^{-1}\widehat{cov}(Z_m,X_k)$, so that $b_{X_k} = (b_{X_{k1}},\ldots,b_{X_{kM}})' =$

$\widehat{cov}(Z, X_m)\widehat{var}(X_m)^{-1}$. Then,

$$\begin{aligned}\widehat{\Sigma}_{\gamma,k_1k_2} &= \Big((\Lambda A_{X_{k_1}}\Lambda)^{\frac{1}{2}}(\Lambda A_{X_{k_2}}\Lambda)^{\frac{1}{2}}\Big)^{-1}\Big[\tau_{k_1k_2} - (\Lambda' b_{X_{k_1}})\Big((\Lambda A_{X_{k_1}}\Lambda)^{\frac{1}{2}}(\Lambda A_{X_{k_2}}\Lambda)^{\frac{1}{2}}\Big)^{-1}(\Lambda' b_{X_{k_2}})\Big] \\ &\xrightarrow{P} \Sigma_{\gamma,k_1k_2},\end{aligned}$$

where the second line follows by WLLN. Finally, $\widehat{\gamma}_k = (\Lambda A_{X_k}\Lambda)^{-1}\Lambda' b_{X_k}$, and $\widehat{\gamma} = (\widehat{\gamma}_1, \ldots, \widehat{\gamma}_K)$.

Proof of Proposition 2

We derive the asymptotic distribution of the statistic $\widehat{T} = F_{k|-k}(L - K + 1)$ under the null hypothesis $H_{0k} : \gamma_k - \gamma_{-k}\delta_0 = 0$ for some unique $\delta_0 \in \mathbb{R}^{K-1}$. Let $\widehat{m}_k(\delta) = \widehat{\gamma}_k - \widehat{\gamma}_{-k}\delta$, $\widehat{\Omega}_k(\delta) = h(\delta)\widehat{\Sigma}_{\gamma,k}h(\delta)'$, and $\Omega_k = h(\delta_0)\Sigma_{\gamma,k}h(\delta_0)'$. Then, under standard GMM arguments, the estimate $\widehat{\delta} = \arg\min \widehat{m}_k(\delta)'\widehat{\Omega}_k(\delta)^{-1}\widehat{m}_k(\delta)$ satisfies the first order expansion

$$\sqrt{n_X}(\widehat{\delta} - \delta_0) = -(M_{-k}'\Omega_k^{-1}M_{-k})^{-1}M_{-k}'\Omega_k^{-1}\sqrt{n_X}\widehat{m}_k(\delta_0) + o_P(1),$$

where $M_{-k} = -\gamma_{-k}$. Then, note that $\sqrt{n_X}(\widehat{m}_k(\widehat{\delta}) - \widehat{m}_k(\delta_0)) = M_{-k}\sqrt{n_X}(\widehat{\delta} - \delta_0) + o_P(1)$. Hence, for $R_k = I_L - \Omega_k^{-\frac{1}{2}}M_{-k}(M_{-k}'\Omega_k^{-1}M_{-k})^{-1}M_{-k}'\Omega_k^{-\frac{1}{2}}$,

$$\Omega_k^{-\frac{1}{2}}\sqrt{n_X}\widehat{m}_k(\widehat{\delta}) = R_k\Omega_k^{-\frac{1}{2}}\sqrt{n_X}\widehat{m}_k(\delta_0) + o_P(1).$$

Now, since $\widehat{T} = n_X\widehat{m}_k(\widehat{\delta})'\widehat{\Omega}_k(\delta)^{-1}\widehat{m}_k(\widehat{\delta})$, and $\widehat{T} - n_X\widehat{m}_k(\widehat{\delta})'\Omega_k^{-1}\widehat{m}_k(\widehat{\delta}) = o_P(1)$ by Proposition 1, we have $\widehat{T} = \mathcal{U}_k'R_k\mathcal{U}_k + o_P(1)$ where $\mathcal{U}_k = \Omega_k^{-\frac{1}{2}}\sqrt{n_X}\widehat{m}_k(\delta_0)$. Thus, $\widehat{T} \xrightarrow{D} \chi^2_{L-K+1}$ since R_k is idempotent of rank $L - (K - 1)$, and from Proposition 1, $\mathcal{U}_k \sim N(0, I_L)$ as $n_X \to \infty$.

Proof of Proposition 3

The oracle GMM estimator which assumes knowledge of κ^2 is given by $\bar{\theta} = \arg\min_\theta \widehat{Q}(\theta, \kappa^2)$, where $\widehat{Q}(\theta, \kappa^2) = \widehat{g}(\theta)'\widehat{\Omega}(\theta, \kappa^2)^{-1}\widehat{g}(\theta)$. Consistency of $\bar{\theta}$ for θ_0 is given by standard GMM arguments; see, for example, Theorem 3.1 of Newey and McFadden (1994).[7] Moreover, by the first order condition, we have $\nabla_\theta\widehat{Q}(\bar{\theta}, \kappa^2) = 0$.

By the mean value theorem, there exists $\dot{\theta} \in \mathbb{R}^K$ on the line segment joining $\bar{\theta}$ and θ_0 such that $\nabla_\theta\widehat{Q}(\theta_0, \kappa^2) + \nabla_{\theta\theta'}\widehat{Q}(\dot{\theta}, \kappa^2)(\bar{\theta} - \theta_0) = 0_{K\times 1}$. By standard GMM arguments, it can be shown that $n_Y\nabla_{\theta\theta'}\widehat{Q}(\dot{\theta}, \kappa^2) \xrightarrow{P} -(\gamma'\Omega^{-1}\gamma)^{-1}$, where $\Omega = \Sigma_\Gamma + I_L\kappa^2 + c\varphi(\theta_0)\Sigma_\gamma\varphi(\theta_0)'$, $\varphi(\theta_0) = \theta_0' \otimes I_L$, and $\sqrt{n_Y}\nabla_\theta\widehat{Q}(\theta_0, \kappa^2) = -\gamma'\Omega^{-1}\sqrt{n_Y}\hat{g}(\theta_0) + o_P(1) \xrightarrow{D} N(0_{K\times 1}, \gamma'\Omega^{-1}\gamma)$. Therefore, by Cramer's theorem, $\sqrt{n_Y}(\bar{\theta} - \theta_0) \xrightarrow{D} N(0_{K\times 1}, (\gamma'\Omega^{-1}\gamma)^{-1})$.

References

[1] Fatima Batool, Ashish Patel, Dipender Gill, and Stephen Burgess. Disentangling the effects of traits with shared clustered genetic predictors using multivariable Mendelian randomization. *Genetic Epidemiology*, 46(7):415–429, 2022.

[2] Eleanor Sanderson, Wes Spiller, and Jack Bowden. Testing and correcting for weak and pleiotropic instruments in two-sample multivariable Mendelian randomization. *Statistics in Medicine*, 40:5434–5452, 2021.

[3] Lars P Hansen, John Heaton, and Amir Yaron. Finite-sample properties of some alternative GMM estimators. *Journal of Business and Economic Statistics*, 14(3):262–280, 1996.

[4] Whitney K Newey and Richard J Smith. Higher order properties of GMM and generalized empirical likelihood estimators. *Econometrica*, 72(1):219–255, 2004.

[5] Lars P Hansen. Large sample properties of generalized method of moments estimators. *Econometrica*, 50(4):1029–1054, 1982.

[6] Sheng Wang and Hyunseung Kang. Weak-instrument robust tests in two-sample summary-data Mendelian randomization. *Biometrics*, 78(4):1699–1713, 2022.

[7] Whitney K Newey and Daniel McFadden. Large sample estimation and hypothesis testing. *Handbook of Econometrics*, 4(1):2111–2245, 1994.
